# Transcranial Direct-Current Stimulation May Improve Discourse Production in Healthy Older Adults

**DOI:** 10.3389/fneur.2020.00935

**Published:** 2020-08-26

**Authors:** Shereen J. Matar, Isaac O. Sorinola, Caroline Newton, Marousa Pavlou

**Affiliations:** ^1^Centre for Human & Applied Physiological Sciences, Faculty of Life Sciences & Medicine, King's College London, London, United Kingdom; ^2^Department of Public Health Sciences, Faculty of Life Sciences & Medicine, King's College London, London, United Kingdom; ^3^Division of Psychology & Language Sciences, Faculty of Brain Sciences, University College London, London, United Kingdom

**Keywords:** language, tDCS, neurorehabilitation, discourse, aphasia

## Abstract

**Background:** The use of transcranial direct-current stimulation (tDCS) for therapeutic and neurorehabilitation purposes has become increasingly popular in recent years. Previous research has found that anodal tDCS may enhance naming ability and verbal fluency in healthy participants. However, the effect of tDCS on more functional, higher level language skills such as discourse production has yet to be understood.

**Aims:** The present study aimed to investigate in healthy, older adults (a) the effect of anodal tDCS on discourse production vs. sham stimulation and (b) optimal electrode placement for tDCS to target language improvement at the discourse level.

**Methods:** Fourteen healthy, older right-handed participants took part in this sham controlled, repeated measures pilot study. Each participant experienced three different experimental conditions; anodal tDCS on the left inferior frontal gyrus (IFG), anodal tDCS on the right IFG and sham stimulation while performing a story telling task. Significant changes in language performance before and after each condition were examined in three discourse production tasks: recount, procedural and narrative.

**Results:** Left and right IFG conditions showed a greater number of significant within-group improvements (*p* < 0.05) in discourse production compared to sham with 6/12 for left IFG, 4/12 for right IFG and 2/12 for sham. There were no significant differences noted between tDCS conditions. No relationship was noted between language performance and physical activity, age, or gender.

**Conclusions:** This study suggests that anodal tDCS may significantly improve discourse production in healthy, older adults. In line with previous tDCS language studies, the left IFG is highlighted as an optimal stimulation site for the modulation of language in healthy speakers. The findings support further exploration of tDCS as a rehabilitative tool for higher-level language skills in persons with aphasia.

## Introduction

The use of transcranial direct-current stimulation (tDCS) for therapeutic and neurorehabilitation purposes has become increasingly popular in recent years. Transcranial direct-current stimulation is a safe non-invasive brain stimulation (NIBS) method that can modify spontaneous cortical activity in targeted brain regions (1, 2). The prolonged effects of NIBS can be inhibitory or excitatory depending on polarity of current flow whereby brain excitability is often increased by anodal tDCS and decreased by cathodal tDCS (2–4). Although the exact mechanisms behind the effect of tDCS are yet to be fully understood, several studies demonstrate its effectiveness in supporting motor and cognitive function recovery, while a growing number have assessed its impact on language (5–8).

Anodal tDCS has been shown to modulate language performance in healthy speakers and in people with aphasia, including improvements in verbal fluency (3, 4, 9, 10) and naming (11–17) during word level language tasks such as picture naming. Few studies though have investigated the impact of tDCS on language performance at the discourse level (18, 19).

Natural verbal communication is seldom in isolated words. Discourse is a higher-level of language expanding beyond simple sentences and allows for the production of meaningful language in everyday situations (20, 21) including recounting information, telling stories or narratives, conversing, and giving instructions. More recently greater focus has been given to this level of language in interventions for people with aphasia. A range of interventions have shown encouraging results for improving discourse production including improved conversational ability, lexical retrieval, and syntactic structure (22–28). However, it remains unclear whether tDCS can be an effective supplementary treatment method in aphasia. Investigating the effect of tDCS on discourse production in healthy speakers can provide insight into its potential for enhancing aphasia treatment outcomes.

The effect of frontal tDCS and significance of the left inferior frontal gyrus (LIFG) in language production has been highlighted in studies assessing single word production, but discourse production is more complex and involves an interaction of linguistic and cognitive processes supported by several brain regions (29, 30). Although regions of the left hemisphere important for language function, including Broca's (IFG) and Wernicke's area, have been identified, the contribution of the right hemisphere to language processing is not well-established (31). Previous studies have found individuals with right hemispheric damage produce narratives with reduced information and organizational aspects compared to healthy speakers (31, 32). Additionally, imaging studies have shown regular responses in both the left and right motor cortices and bilaterally in the IFG during discourse production (29, 30). These findings support the involvement of the right hemisphere in discourse production. Thus, when investigating the effect of tDCS at discourse level, the right hemisphere must be considered and the LIFG cannot be assumed to be the optimal stimulation site. To date though no studies have compared the effect of left and right hemispheric tDCS on discourse speech production.

Thus, the aims of the present study were to investigate in healthy older adults the (a) effect of anodal tDCS on discourse speech production vs. sham and (b) optimal electrode placement for tDCS to target language improvement at discourse level. This study recruited healthy, older participants to be more representative of the median age of the stroke population which is 77 years old (33). Since psychological well-being, higher physical activity, and social participation levels have been linked to improved cognitive function (34–37), this study also investigated if there was a relationship between participants' language performance and these three factors. The null hypothesis assumed no significant difference between anodal tDCS and sham, whereas the alternative hypothesis assumed that all anodal tDCS conditions would result in greater language modulations and improvements in discourse production compared to sham. Changes in discourse production were expected to differ depending on electrode placement site, revealing a more optimal site for tDCS stimulation for improving discourse language skills in future studies.

## Methods

### Design

This was a sham controlled, single blinded (participant) randomized repeated measures pilot tDCS study. The protocol was approved by the King's College London Research Ethics Review Board. Written informed consent was obtained from each participant. All study procedures were in accordance with the Declaration of Helsinki (38).

Each participant experienced three different experimental conditions in random order: anodal tDCS on LIFG, anodal tDCS on right IFG (RIFG), and sham. To control for order and learning effects, conditions were tested in a counterbalanced order across subjects using a Latin Square design. Participants attended four individual sessions each lasting ~60 min. Sessions were arranged 2-weeks apart in order to decrease both the impact of carryover effects from the previous sessions and statistical bias (39, 40). The final 2-week follow-up assessment was performed during the fourth session.

### Participants

Fourteen healthy, community-dwelling, independently mobile, older participants (9 females and 5 males: age range = 65–79, *M* = 73 years) were recruited between September and November 2018. Participant inclusion criteria were healthy, older adults who were ≥65 years old in order to match the current median age of persons with stroke in the UK (33), native English-speaking, right-handed, normal aided, or unaided visual acuity, and at least a secondary school education level. Exclusion criteria were history of neurological disease or cognitive impairment, seizures, implanted metal, or any other tDCS contraindications (2). All potential participants completed a pre-tDCS screening questionnaire to confirm study eligibility. Collected information included past medical history [i.e., any neurological, psychiatric, and/or cognitive diagnoses or symptoms; (2, 9, 41, 42)] and hand use preference in activities such as writing and eating to determine handedness (adapted from the Edinburgh Handedness Inventory) (43).

### Intervention and Blinding

#### tDCS Stimulation

**Figure 1:**
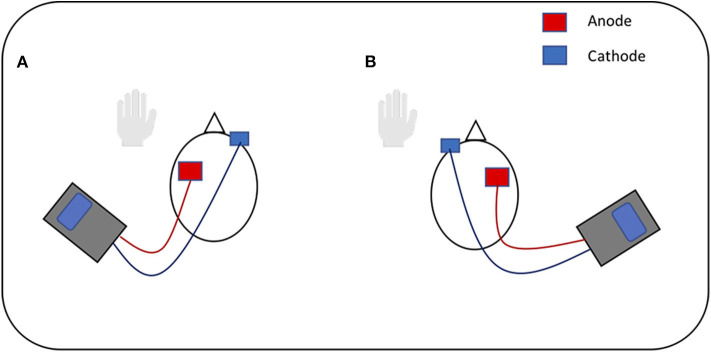
Visual representation of tDCS. **(A)** Anodal stimulation of left inferior frontal gyrus: anode over the left IFG and cathode over the right supraorbital ridge. **(B)** Anodal stimulation of right inferior frontal gyrus: anode over the right IFG and cathode over the left supraorbital ridge. Figure adapted from Figure 1 in (48), licensed under the Creative Commons Attribution License 4.0.

This study followed previous protocols used in tDCS language studies (3, 15, 18, 44) as replication is encouraged to support the development of common experimental guidelines for behaviors, including language production, and allow for more efficient comparisons between tDCS studies (2). Three stimulation conditions were tested: anode over the LIFG, anode over the RIFG and sham. In line with safe guidelines (2), anodal stimulation was produced through a battery operated constant current stimulator (DC-Stimulator Plus, NeuroConn, Ilmenau, Germany). A pair of saline soaked sponge (5 × 7 cm) electrodes were used to deliver the stimulation to the target area. As noted in previous studies which stimulated the IFG (15, 45, 46), the 10-10 EEG system (47) was used as a guide for electrode placement and the stimulation electrode was placed over FC5 for LIFG and FC6 for RIFG stimulation. The cathode electrode for each condition was placed on the contralateral supraorbital ridge [Figure 1, adapted from (48)]. A constant current of 2 mA was applied for 20 min at the beginning of each session. This intensity level and duration has been found to be effective in modulating language production in healthy speakers (3, 44). During sham condition the procedure and electrode placement was identical except that tDCS was switched off after 30 s. A ramping period of 30 s was applied at the beginning and end for every condition. The “study mode” option on the tDCS stimulator blinded participants to the tDCS condition (3, 10, 14).

### Outcome Measures

#### Language Tasks

Participants attended four sessions in total: three for tDCS application and one final fourth session for 2-week follow-up testing. Sessions one to three each began with baseline language testing and ended with post-treatment testing immediately after tDCS application. Pre-post-treatment testing included production of three different discourse language tasks:

1. Recount (i.e., describing a previous holiday)
2. Procedural (i.e., describing the steps of how to make scrambled eggs) (49)
3. Narrative [i.e., retelling the Cinderella story (50)].

The same tasks were used for all participants and conditions. Participants were only provided with simple instructions for all discourse language tasks (i.e., “tell me about a past holiday”). All pre post-testing discourse language samples were recorded using a small audio recorder (TF-85, Homder, China) and orthographically transcribed for analysis.

Each tDCS application was performed with a concurrent story-telling task. Participants were presented with the wordless picture book, Frog, Where Are You (51), and asked to verbally produce a story narrative related to the pictures (29). Before beginning, participants were asked to “imagine they are telling the story to a child, providing as much detail as possible” (29).

#### Discourse Production Measures

Language samples from each task (recount, procedural and narrative) were analyzed for quantity and level of informativeness. The Quantitative Production Analysis [QPA (21, 52)] protocol was used to extract the discourse sample and measure the number of discourse words. Applied measures were based on previous studies which involved similar language samples (53–57) and included word total, verb total, utterance total, and percent Correct Information Units (%CIU). Based on the QPA coding protocol, utterances were considered sentences if they included a predicate-argument structure (58). Verb retrieval within discourse or the process of formulating a spoken verb from a concept was measured using verb total or the count of all verb productions in a sample (23). Nicholas and Brookshire's %CIU was used to measure the level of informativeness in each sample and was calculated by dividing the number of CIUs by the total number of words in a language sample. Words which were accurate and provided relevant information to the language task were included in the CIU word count (59, 60).

The following questionnaires were completed by all participants to assess for correlations between language task performance with participants' psychological wellbeing, physical activity and participation levels. The Hospital Anxiety and Depression Scale (HADS) is a 14-item scale that assesses anxiety (HAD-A) and depression (HAD-D) symptoms; scores range between 0 and 21 for both anxiety and depression subscales, where a score between 8 and 11 indicates a borderline case and a score ≥11 indicates anxiety or depression (61, 62). The EPIC Physical Activity Questionnaires (EPAQ2) is a reliable and valid self-completed questionnaire which collects information on an individual's physical activity at home, at work and recreation. Based on total activity hours in the last 12 months, the physical activity index is applied to categorize an individual's levels of physical activity into “inactive,” “moderately inactive,” “moderately active,” and “active” (63). The Keele Assessment of Participation (KAP) is a brief questionnaire which measures an individual's level of participation in various activities including activities of daily living, work, and social activities (64, 65).

### Statistical Analysis

Data analysis was conducted using SPSS 25 (IBM Inc.) and Prism 8 (GraphPad Software, Inc.). Kolmogorov-Smirnov tests assessed normality and non-parametric tests were utilized in cases where normality was not met. For each tDCS condition (LIFG, RIFG, and sham) a Wilcoxon signed-ranks test was performed using false detection rate adjusted *p*-values (66) to identify significant pre-post-stimulation changes in the three language tasks for each measure. The Friedman test determined pre-stimulation baseline changes between tDCS conditions. Spearman rho tests were used to identify any relationship between language performance across tDCS conditions, participants' demographic data, and questionnaire results. The significance level was *p* ≤ 0.05 for all tests.

## Results

Fourteen healthy, older adults were recruited (mean age = 73 ± 5; Females = 9, Males = 5). Participant demographics are presented in Table 1.

**Table 1:** Demographic data and questionnaire results for each participant.

**Participant**	**Age range**	**HAD-A**	**HAD-D**	**EPAQ**	**KAP**
	**(*****n*** **=** **14)**	**(*****n*** **=** **11)**	**(*****n*** **=** **11)**	**(*****n*** **=** **11)**	**(*****n*** **=** **11)**
P01	76–80	2	0	4	0
P02	71–75	6	5	3	0
P03	65–70	4	1	2	0
P04	76–80	DNC	DNC	DNC	DNC
P05	71–75	3	2	4	0
P06	76–80	5	3	2	0
P07	71–75	2	1	4	0
P08	65–70	5	8	2	0
P09	76–80	5	3	4	0
P10	76–80	0	0	3	0
P11	71–75	DNC	DNC	DNC	DNC
P12	65–70	5	1	2	0
P13	65–70	7	0	4	0
P14	71–75	DNC	DNC	DNC	DNC
Mean (*SD*)	73 (5)	4 (2)	2 (2)	3 (1)	0

*DNC, did not complete; HADS, Hospital Anxiety and Depression Scale (67); EPAQ, EPIC Physical Activity Questionnaire (63); KAP, Keele Assessment of Participation (65). EPAQ physical activity index: 1 = inactive, 2 = moderately inactive, 3 = moderately active, 4 = active. KAP scoring: 0 = no participation restrictions, 1–11 = indication of restriction in participation in at least one activity*.

All participants tolerated tDCS. No adverse reactions were reported or observed. All participants attended 100% of sessions.

### tDCS and Language Task Performance

#### Pre-stimulation Assessment

No significant differences between tDCS conditions were noted in baseline pre-stimulation performances in each language task for all discourse measures.

#### Word Total

No significant differences between tDCS conditions were noted for word total improvements in all language tasks.

**Figure 2:**
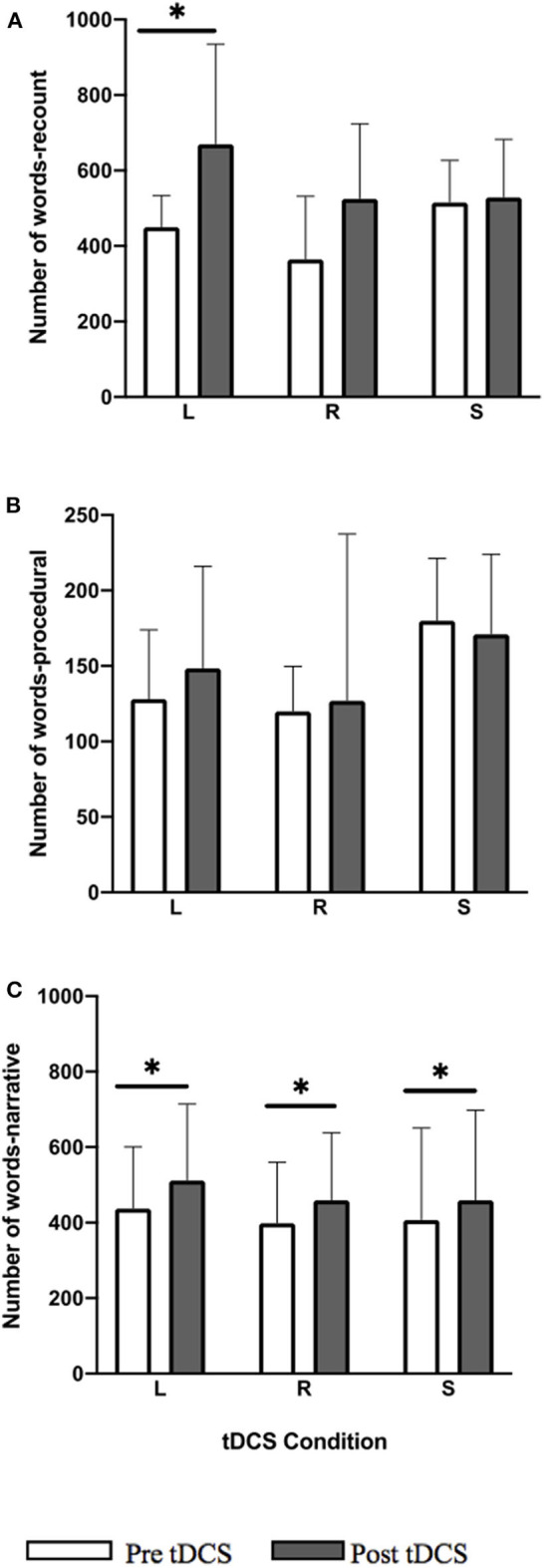
Pre-post-stimulation group changes in word total (median values and interquartile range) in **(A)** recount, **(B)** procedural, and **(C)** narrative. Significant within-group differences (*p* ≤ 0.05) using false detection rate adjusted *p*-values are indicated by a (*). tDCS conditions are LIFG (L), RIFG (R), and sham (S).

All three tDCS conditions showed a significant within-group improvement in word total for the narrative task (Figure 2; LIFG: *Z* = −3.045, *p* = 0.006; RIFG: *Z* = −3.297, *p* = 0.005; sham: *Z* = −2.417, *p* = 0.036). In the LIFG condition 13/14 participants showed a positive change (i.e., improvement) and 1/14 performed worse; in RIFG condition 14/14 showed positive change; and in sham 11/14 showed positive change and 3/14 performed worse. A significant within-group improvement in recount was noted only for the LIFG condition (Figure 2; *Z* = −3.296, *p* = 0.005) where all 14 participants showed an improvement post-stimulation.

Improvements for the narrative task were maintained at 2-week follow-up for LIFG (follow-up vs. pre-stimulation *p* = 0.034) and RIFG (*p* = 0.028) conditions.

#### Utterance Total

No significant differences between tDCS conditions were noted for improvements in utterance total in all language tasks. For procedural, the between-group difference approached significance (*p* = 0.062) where RIFG had a greater number of utterances post-stimulation compared to LIFG and sham.

**Figure 3:**
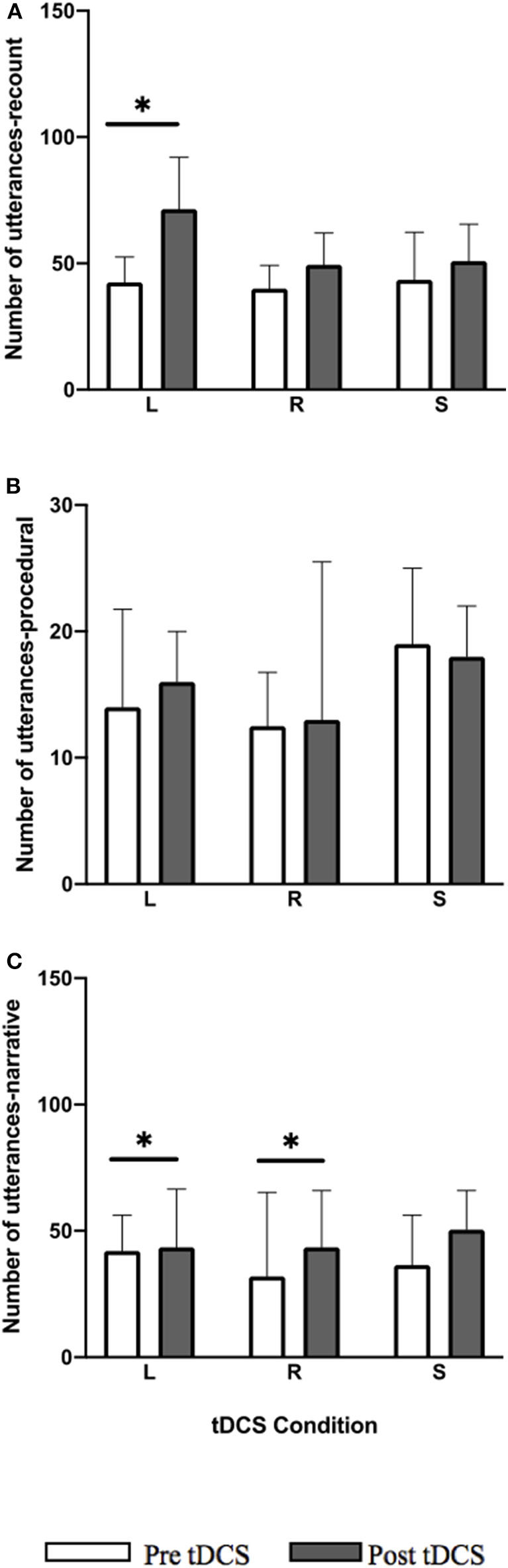
Pre-post-stimulation group changes in utterance total (median values and interquartile range) in **(A)** recount, **(B)** procedural, and **(C)** narrative. Significant within-group differences (*p* ≤ 0.05) using false detection rate adjusted *p*-values are indicated by a (*). tDCS conditions are LIFG (L), RIFG (R), and sham (S).

A significant pre-post-treatment change was noted with within-group improvements in utterance total for both the recount (Figure 3; *Z* = −3.297, *p* = 0.009) and narrative (Figure 3; *Z* = −2.657, *p* = 0.024) tasks in LIFG condition. In recount 14/14 participants showed positive change and in narrative 11/14 showed positive change, 1/14 no change, and 2/14 performed worse. For the RIFG condition, a significant within-group improvement was noted only for the narrative task (Figure 3; *Z* = −2.794, *p* = 0.0225) where 12/14 participants showed positive change and 2/14 performed worse. No significant improvements were noted in any of the tasks for the sham condition. Within-group improvements noted for LIFG and RIFG conditions were not maintained at follow-up.

#### Verb Total

No significant differences between tDCS conditions were noted for verb total improvements in all language tasks.

**Figure 4:**
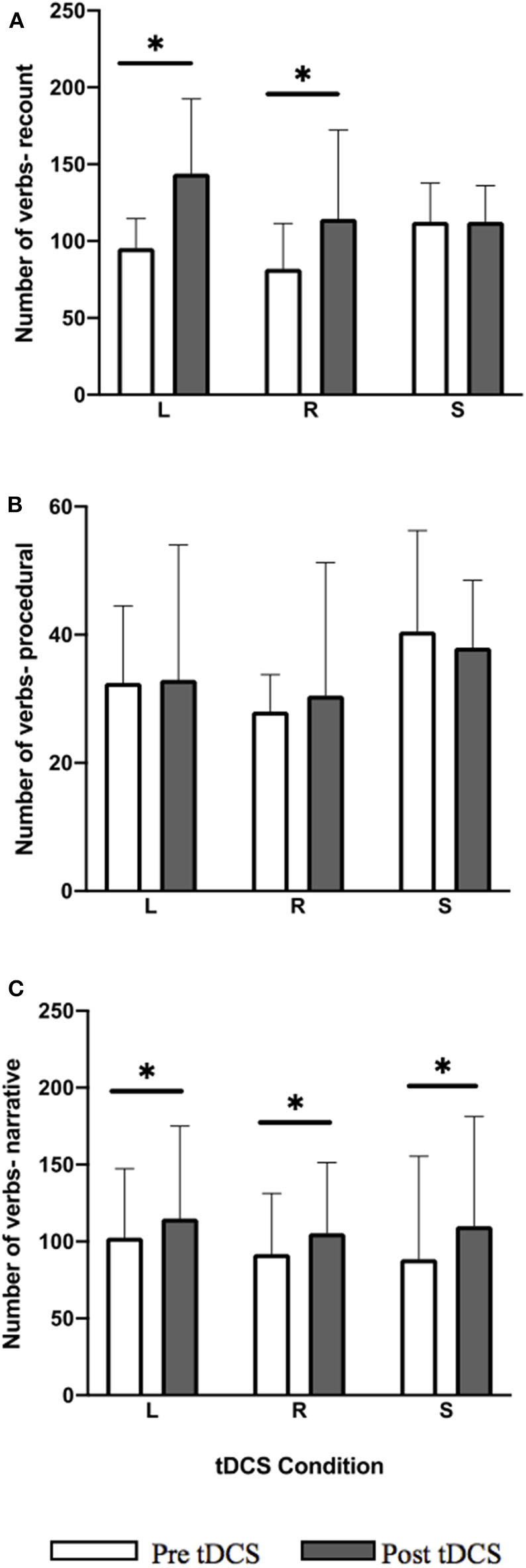
Pre-post-stimulation group changes in verb total (median values and interquartile range) in **(A)** recount, **(B)** procedural, and **(C)** narrative. Significant within-group differences (*p* ≤ 0.05) using false detection rate adjusted *p*-values are indicated by a (*). tDCS conditions are LIFG (L), RIFG (R), and sham (S).

For the LIFG and RIFG conditions the total number of verb showed significant within-group improvements for the recount (Figure 4; LIFG: *Z* = −3.297, *p* = 0.0045; RIFG: *Z* = −2.229, *p* = 0.0468) and narrative (Figure 4; LIFG: *Z* = −3.048, *p* = 0.006; RIFG: *Z* = −3.297, *p* = 0.0045) language tasks. In the recount task 14/14 participants showed positive change in LIFG condition, and 11/14 showed positive change and 3/14 performed worse in RIFG condition. In the narrative task for LIFG condition 12/14 showed positive change and 2/14 performed worse and for the RIFG condition 14/14 showed positive change. For sham, post-stimulation within-group improvements in the narrative task were significant (Figure 4; *Z* = −2.726, *p* = 0.014) where 11/14 showed positive change, 1/14 showed no change, and 2/14 performed worse. Within-group improvements were not maintained at follow-up.

#### %CIU

**Figure 5:**
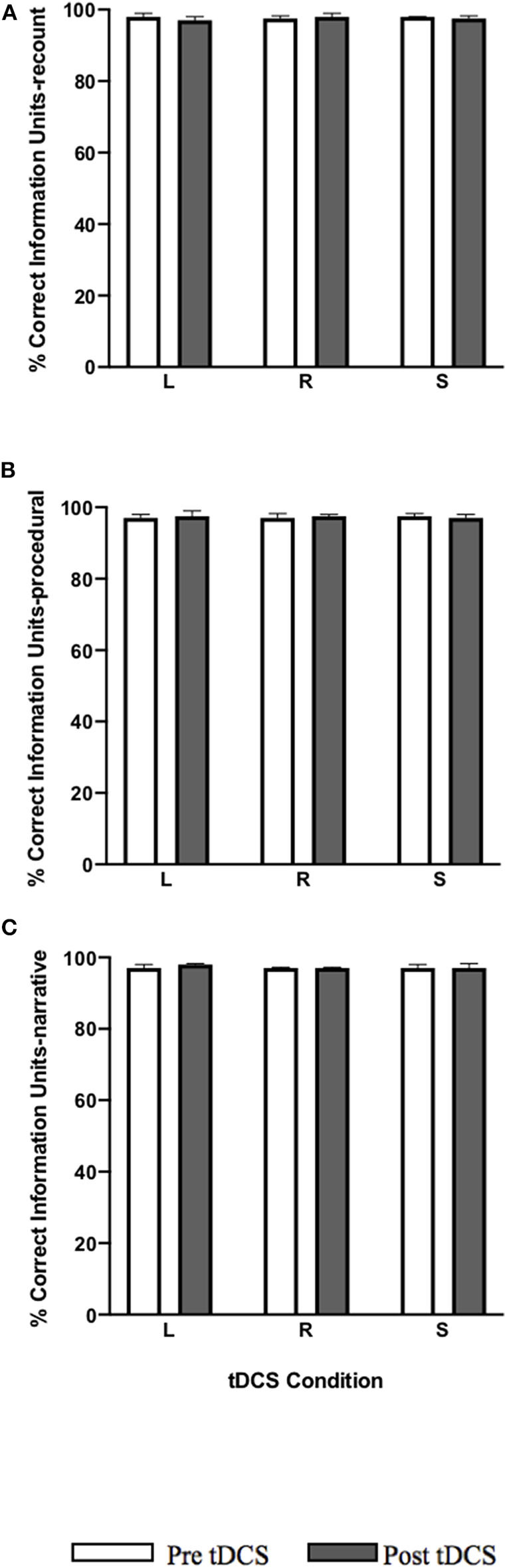
Pre-post-stimulation group changes in %CIU (median values and interquartile range) in **(A)** recount, **(B)** procedural, and **(C)** narrative. tDCS conditions are LIFG (L), RIFG (R), and sham (S).

No significant pre-post-treatment change was noted in %CIU in all tDCS conditions for each language task: recount, procedural, and narrative (Figure 5).

### Questionnaire Results and Correlations

Eleven participants completed the HADS, EPAQ, and KAP (descriptive data are presented in Table 1). Participants' (*n* = 11) scores were within normal ranges for the HAD-D and HAD-A except for a single participant with a HAD-A score of 8/21 indicating a borderline case. EPAQ scores identified an average “moderately active” level of physical activity across participants with none in the inactive range, four in the moderately inactive range, two in the moderately active range, and five in the active range. On the KAP, all participants (*n* = 11) scored 0/11, indicating no restrictions for daily participation.

Spearman rho tests revealed no significant correlations between physical activity levels, HADS scores, age, or gender and performance in language tasks across tDCS conditions.

## Discussion

The aim of this study was to investigate the effect of anodal tDCS on discourse production and optimal electrode placement for the enhancement of language at this level in healthy older speakers. Several discourse measures were used as this is encouraged in aphasia research in order to gain a comprehensive analysis at the level of discourse speech and identify areas of strength in discourse output (20, 68, 69). Overall, the results demonstrate a greater number of significant within-group language modulations for both anodal tDCS conditions compared to sham, where the greatest improvements in language performance were noted for LIFG stimulation. The stimulation effect was not equal across discourse language tasks and there was no relationship found between language performance and psychological well-being, physical activity, age, or gender. The above main findings will be further discussed below.

Study findings demonstrate that both active anodal tDCS conditions (LIFG and RIFG) result in a greater number of significant within-group improvements in language performance than sham (6/12 for left IFG, 4/12 for right IFG, and 2/12 for sham). Both anodal tDCS on LIFG and RIFG produced significant within-group improvements in recount and narrative language tasks, whereas improvements resulting from sham stimulation were limited to the narrative task. Previous work found that repeating narrative tasks may lead to some improvements in language production due to increased familiarity from using identical narrative elicitation methods (70). Therefore, although there was a 2-week washout period between sessions, the small number of significant within-group improvements in narrative noted with sham stimulation may be related to practice effects. In line with a recent review (44), these findings support the use of anodal tDCS as an effective technique for modulating language production in healthy older individuals. Previous tDCS studies in healthy speakers have demonstrated improvements within word level tasks (5, 11, 13, 16), however, we believe this is the first study indicating that the spontaneous neuron activity and cortical excitability changes caused by tDCS (71) could also enhance word retrieval and overall language performance within discourse of healthy speakers, a more complex and functional form of language production.

Most previous tDCS studies have focused on examining the impact of tDCS in younger adults. However, normal physiological aging results in a decline in language abilities, including word retrieval, due to changes in synaptic connectivity which may alter neuroplasticity mechanisms (12). The current findings are the first to indicate that tDCS may have a positive effect on higher-level language performance in healthy older adults and may be an effective method to counteract age-related changes in language abilities.

Although our findings support past imaging studies (29, 72) demonstrating an important role of the right hemisphere, including the RIFG, in discourse production, significant improvements were noted for a greater number of factors in the LIFG (6/12) compared to RIFG (4/12) condition. In some areas the RIFG condition showed similar improvements to LIFG however more improvement in the number of words and utterances in the recount task were noted only with LIFG, suggesting it as a more optimal stimulation site for improving discourse production. No significant differences were noted between tDCS conditions which may be due to the study's small sample size. Our findings are in accordance with previous research supporting the importance of the LIFG for language production of words and sentences (73, 74). Although the exact mechanisms underpinning tDCS and neuroplasticity are not clearly understood, recent functional magnetic resonance imaging (fMRI) work has sought to analyse the neural mechanisms which underlie behavioral improvements resulting from tDCS (75). Studies which combined tDCS with fMRI found that compared to sham, anodal tDCS on LIFG during different word level language tasks (i.e., naming, verb learning) decreases brain activity (15, 76, 77) and modulates language network connectivity in healthy speakers (45, 76, 77). Neural changes (i.e., reduced activity in LIFG) which are associated with improved behavior suggest that tDCS indirectly improves performance by promoting increased processing efficiency (45, 76). The number of significant improvements resulting from anodal tDCS on LIFG in this study may have resulted from neuroplastic aftereffects caused by similar neural mechanisms. However, more work is needed in this area including examining higher level language output such as discourse to better understand the neural mechanisms which allow tDCS to modulate language performance at this level (73).

The tDCS effect was not equal across the three discourse tasks. The majority of significant within-group improvements resulting from both active tDCS conditions were noted in recount language samples. Significant improvements in narrative language samples were noted in all three tDCS conditions, suggesting that tDCS has a greater impact on recounting discourse skills. No significant changes were observed for the procedural task across conditions. This discrepancy may be directly related to the language task completed during tDCS stimulation. It has been previously found that the task completed during stimulation can impact the effects of tDCS on post-stimulation performance (78), where greater post-stimulation tDCS effects are observed in tasks which require similar cognitive abilities to the one completed during stimulation. In a previous study, participants who received tDCS while completing a picture naming task (nouns) had post-stimulation improvements only at the word level in the number of nouns produced, indicating that the task completed during tDCS may have promoted the production of nouns (79). In the current study the concurrent story telling task, may have supported improvements in the similar post-stimulation recount and narrative tasks which also involves the description of a sequence of events and actions that develop over time (80), but not the procedural task as no similar activities were performed during tDCS stimulation. Further studies are required to understand the specific influence of the concurrent task on post-tDCS effects.

Increasing physical activity has been found to improve cognitive function and promote neuroplasticity (35), however, the current study did not identify an association between physical activity levels and language performance. It is important to note though that the majority of participants were in the active range and none were categorized as inactive. Anxiety has been previously associated with deficits in communication (36) and depression has been linked to cognitive dysfunction (34). Since participants in this study had normal range psychological symptoms except for a single participant with a borderline caseness of anxiety, it was not possible to properly examine the association between psychological symptoms and language performance.

Brain activation in males may be lateralised to the LIFG whereas in females activation occurs bilaterally in both the left and right IFG during phonological language tasks (81, 82). No significant association was observed though between gender and language performance post-stimulation on RIFG and LIFG. This may be due to the more complex nature of discourse production which requires activation of both hemispheres regardless of gender. Similarly, no significant relationship was found between age and pre-post-stimulation change in language performance in this cohort of healthy older adults. Since the mean age of participants was 73 years (range = 65–79), a group with a wider age range may have performed differently and have enabled us to identify an association. However, this is a pilot study and further work is required to establish if there is a relationship between discourse language performance and psychological state, physical activity, age, and gender in healthy and patient populations.

The use of sham condition to control for non-specific tDCS effects provides confidence that our results were due to anodal tDCS. There were a number of study limitations though. The small sample size reduced statistical power; electrode placement may have reduced placement accuracy as it depends on human measurement using the 10-10 EEG system as a guide; despite the 2-week washout period between sessions there may have been carryover effects; and non-blinding of the main researcher and outcome assessor to the tDCS condition introduces bias. As this study did not simultaneously investigate excitability of the stimulated frontal regions, future work incorporating imaging techniques would strengthen the hypotheses made regarding the effects of tDCS on discourse production. Additional investigations of the mechanism of tDCS in the LIFG and other language related cortical areas may establish the tDCS induced neurophysiological changes that are responsible for improving language production. Comparing the effect of tDCS on LIFG with other tDCS montages such as bi-hemispheric stimulation (i.e., bilateral IFG stimulation) still needs to be explored to confirm the optimal tDCS target for improving language production at discourse level. Finally, this study followed standard tDCS methods which typically use electrode sizes of 5 × 7 cm or 5 × 5 cm, however, a more focal method, HD-tDCS, which uses small ring electrodes, may provide a stronger understanding of the effects of IFG stimulation on discourse production (2).

## Conclusion

This study demonstrates that anodal tDCS may significantly improve discourse production in healthy, older adults, and further reinforces the LIFG both as a critical region for language production and as an optimal stimulation site for the modulation of language in healthy speakers. Based on the study findings, although both LIFG and RIFG conditions produced improvements in discourse production, significant within-group improvements were greater for the LIFG condition. These findings contribute to the foundation for future clinical trials investigating the effects of tDCS on discourse production and support the use of tDCS as a rehabilitative tool for higher-level language skills in people with aphasia due to neurological conditions.

## Data Availability Statement

The raw data supporting the conclusions of this article are available on request to the corresponding author.

## Ethics Statement

The studies involving human participants were reviewed and approved by King's College London Biomedical & Health Sciences, Dentistry, Medicine and Natural & Mathematical Sciences Research Ethics Review Board. The patients/participants provided their written informed consent to participate in this study.

## Author Contributions

SM, IS, and MP: study concept. SM, IS, CN, and MP: development of methodology, manuscript draft revisions, and editing. SM: data collection and original draft preparation. SM and MP: data analysis. MP: supervision. All authors read and approved the final manuscript.

## Conflict of Interest

The authors declare that the research was conducted in the absence of any commercial or financial relationships that could be construed as a potential conflict of interest.
